# Reduced greenhouse gas mitigation potential of no-tillage soils through earthworm activity

**DOI:** 10.1038/srep13787

**Published:** 2015-09-04

**Authors:** Ingrid M. Lubbers, Kees Jan van Groenigen, Lijbert Brussaard, Jan Willem van Groenigen

**Affiliations:** 1 Department of Soil Quality, Wageningen University, PO BOX 47, 6700AA Wageningen, the Netherlands; 2 Center for Ecosystem Science and Society, Department of Biological Sciences, Northern Arizona University, Flagstaff, Arizona 86011, USA

## Abstract

Concerns about rising greenhouse gas (GHG) concentrations have spurred the promotion of no-tillage practices as a means to stimulate carbon storage and reduce CO_2_ emissions in agro-ecosystems. Recent research has ignited debate about the effect of earthworms on the GHG balance of soil. It is unclear how earthworms interact with soil management practices, making long-term predictions on their effect in agro-ecosystems problematic. Here we show, in a unique two-year experiment, that earthworm presence increases the combined cumulative emissions of CO_2_ and N_2_O from a simulated no-tillage (NT) system to the same level as a simulated conventional tillage (CT) system. We found no evidence for increased soil C storage in the presence of earthworms. Because NT agriculture stimulates earthworm presence, our results identify a possible biological pathway for the limited potential of no-tillage soils with respect to GHG mitigation.

Anthropogenic emissions of the three principal GHGs lead to increased radiative forcing of the atmosphere and are considered the cause of climate change^1^. Soils are a major GHG source, producing approximately one fifth of global CO_2_ emissions^2^, roughly one third of global CH_4_ emissions and two thirds of N_2_O emissions^3^. Agricultural soils are responsible for more than 70% of human-induced N_2_O emissions^1^, but are typically minor emitters of CH_4_, flooded soils used for rice production excluded^4^.

Carbon sequestration in agro-ecosystems is intended to restore previously lost soil organic carbon (SOC) stocks and to reduce soil CO_2_ emissions^5^. Management options such as NT or reduced tillage are often identified as particularly promising tools to achieve this^5^,^6^. However, such practices can influence non-CO_2_ GHG emissions. Soil N_2_O emissions from NT systems have been reported to decrease^7^,^8^, to be unaffected by^9^,^10^, or to increase relative to those from CT systems^11^,^12^,^13^. Production and emission of N_2_O is the result of many interacting biogeochemical processes, making it difficult to predict the effects of different tillage practices. On the one hand, lower temperatures, more aggregated soil structure and less compact soils in NT than CT may reduce N_2_O emissions^14^. On the other hand, larger SOC and higher soil moisture and mineral N content in NT may favour emissions of N_2_O^15^.

The literature on GHG emissions from NT *vs*. CT systems does not consider the possible influence of soil biota on these emissions^16^. Yet, many studies found that tillage management impacts soil biota, such as earthworms, resulting in increased earthworm diversity and -abundance under NT relative to CT^17^,^18^. This is important, because earthworms can affect the GHG balance of soil as well. On the one side, earthworms have been suggested to stimulate carbon sequestration^19^. On the other side, by burrowing and feeding on crop residues or SOC, earthworm activity directly affects many physicochemical soil factors, which in turn affect GHG emissions^20^,^21^. Indeed, multiple experimental studies have now demonstrated that earthworms are capable of increasing N_2_O emissions^22^,^23^, with studies reporting up to 13-fold increases in N_2_O emissions due to the presence of earthworms^23^.

The assessment of earthworm effects on the GHG balance of soils is complicated for several reasons. First, earthworm species can be divided into three functional groups based on the ecological strategies that describe their feeding and burrowing activities: epigeic, anecic and endogeic^24^. These functional groups have been shown to differentially affect N_2_O emissions, depending on, among others, the placement of crop residues within the soil profile^22^. Second, earthworm activity may affect the emission of individual GHGs on different time scales. Lubbers *et al.*^16^ pointed out that the positive effect of earthworms on CO_2_ emissions becomes smaller over time, whereas the effect of earthworms on N_2_O emission increases^16^. Because most experimental studies were performed over a short time scale (<200 days; usually <100 days), the long-term effects of earthworm activity on the soil GHG balance of NT and CT systems are unclear.

Here, we quantified the effect of earthworm presence on the GHG balance of simulated NT systems (with crop residues applied to the surface) *vs.* CT systems (with crop residues ploughed in manually). To do this, we measured N_2_O and CO_2_ emissions and SOC contents in a full factorial 750-day mesocosm experiment, the longest manipulative earthworm-GHG emission study to date (Methods). The global warming potential (GWP) of the simulated NT and CT systems could be calculated by transforming CO_2_ and N_2_O values to CO_2_ equivalents^25^.

Mesocosms (30 cm height, 19.5 cm inner diameter) filled with loess (*Gleyic Luvisol*) soil were supplied with maize (*Zea mays* L.) residue at an application rate of 5 Mg dry matter ha^−1^ every 190 days (in total four times)^26^. Our experimental approach allowed us to strictly control C inputs (no soil C input through plants) and thoroughly measure C outputs (no leaching of SOC), and thus to determine changes in SOC by carrying out straightforward calculations with C inputs and outputs. Earthworms were added at a rate of 125 individuals m^−2^ of the epigeic *Lumbricus rubellus* (Hoffmeister) and/or 225 individuals m^−2^ of the endogeic *Aporrectodea caliginosa* (Savigny), which are representative densities for these earthworm species in agro-ecosystems^17^. The experimental timeline and mesocosm design are shown in Fig. 1.

## Results

### Earthworm effects on cumulative GHG emissions

Earthworm presence increased cumulative residue-induced GHG emissions, expressed in terms of GWP, irrespective of tillage treatment (Fig. 2; *P* < 0.001). On average, the CT treatments increased GHG emissions relative to the NT treatments (*P* < 0.001), but the earthworm effect on GHG emissions was greater in NT treatments throughout the experiment (Table 1 and 2). Earthworms increased GHG emissions only by 7–16% in the simulated CT system, but by 31–42% in the simulated NT system. The higher effect in the NT system was for a large part due to the high relative increase in residue-induced N_2_O emissions by earthworms (+528% for N_2_O and +25% for CO_2_; Fig. 2). Table 1 (model I) shows that the effect of earthworm presence on cumulative N_2_O emissions was not significant after 197 days, but became increasingly significant over time. The CT treatment on the other hand increased cumulative N_2_O emissions only after 197, 378 and 575 days; over time the effect of CT on cumulative N_2_O emissions became less strong and eventually disappeared (Table 1 and Fig. 3). These findings suggest that earthworms could be responsible for much of the often reported increase in N_2_O emissions from NT systems^12^, where residues are typically left on the soil surface and where earthworm populations are typically larger than in CT systems^17^.

In each subsequent period after residue application, the effect of earthworms on cumulative CO_2_ and N_2_O emissions became more distinct (Table 1 and Fig. 3). The effect of both earthworm species on N_2_O emissions became larger over time, suggesting that it is a non-transient effect. The increasing earthworm effect on N_2_O emissions over time, which was predicted by an earlier meta-analysis^16^, has now been shown for the first time in a multi-year study.

### Earthworm species effects in simulated NT and CT systems

Both *L. rubellus* and *A. caliginosa* increased N_2_O and CO_2_ emissions from the simulated NT system throughout the 750 days, but *L. rubellus* generally more so than *A. caliginosa* (Table 2). However, in the simulated CT system, *L. rubellus* did not affect GHG emissions at all, whereas *A. caliginosa* increased emissions of CO_2_ and the GWP (Fig. 2 and Table 2). These findings can be explained by the difference in feeding strategies between the earthworm species. *L. rubellus* feeds mostly on crop residues placed on the soil surface^24^, and is therefore likely to be most active in the topsoil of NT systems. Conversely, *A. caliginosa* feeds mostly on soil organic matter (or incorporated crop residues^22^), and is expected to be more active in the top- and subsoil of CT systems.

### No evidence for increased C storage in the presence of earthworms

Several short-term studies concluded that endogeic earthworms can promote C sequestration by increasing the decomposition of new C input, thereby increasing the amount of stable C, and aiding soil C storage in the long term^19^,^27^,^28^,^29^. However, in our study, which lasted more than 30 times longer than these short-term studies and comprised four residue additions, we found no evidence for increased soil C storage in the presence of earthworms. In fact, soil organic C content in the NT_RC_ treatment was not different from the CT_0_ treatment (Fig. 4), suggesting that the presence of earthworms can reduce the buildup of SOC in NT systems to equal levels as in CT systems. Moreover, the presence of *A. caliginosa* in CT treatments caused the SOC contents to become even smaller. Thus, our findings do not support the assertion that earthworms can promote C storage in the long term. Our findings corroborate the results of medium-term earthworm studies; the endogeic earthworm species *Pontoscolex corethrurus* (Müller, 1856) has been reported to decrease the C content in mesocosms after 5 months^30^, whereas *Octolasion tyrtaeum* (Savigny) increased total CO_2_ production after 150 days^31^.

## Discussion

To determine which earthworm treatments are most representative for realistic CT and NT systems, the impact of tillage on earthworm populations should be taken into account. Ploughing in CT systems can reduce overall earthworm abundance by 60%, but endogeic species such as *A. caliginosa*, may increase five times in biomass after tillage^17^. Therefore, we consider the CT treatments with just *A. caliginosa* or without any earthworms to be the most representative of CT conditions. In NT systems, on the other hand, earthworm abundances are typically 2–9 times greater than for CT systems, and earthworm populations are likely to include both epigeic and endogeic species^17^. Thus, we consider the NT treatment with both earthworm species to be the most representative for NT conditions. When comparing these treatments (NT_RC,_ CT_0_ and CT_C_, marked with rectangles in Fig. 2), earthworms in NT treatments increase the GWP to the same level as in CT treatments, and are likely to offset most reductions in radiative forcing achieved by NT management.

Although the soil type, earthworm densities and soil moisture content used in this study are representative for large areas of cropland in temperate climates (see Methods), they cannot represent all of the world’s arable land. The extent to which NT practices stimulate earthworm presence relative to CT depends on cropping system, climate, and other environmental variables^17^,^18^. As such, the extent to which earthworms reduce the greenhouse mitigation potential of NT practices likely depends on these variables as well (Fig. 2). Thus, to extrapolate the effect of earthworms on the GHG mitigation potential of NT systems in general, our results need to be confirmed for other cropping systems and climatic conditions.

Although we did not include growing plants in our experimental design, it is unlikely that doing so would have changed the conclusions of our study. Because NT practices generally decrease primary production compared to CT systems^32^, plant presence would only increase the effect of NT on the net GWP of the soil. The activity of earthworms generally has a positive influence on plant growth^33^. However, in an experiment with growing plants, Lubbers *et al.*^34^ showed that the stimulating effect of earthworms on plant production did not negate earthworm-induced increases in GHG emissions.

One other aspect that sets our mesocosm study apart from field studies is that soil moisture contents were kept constant throughout the experiment. Indeed, NT practices have been reported to increase soil moisture contents under field conditions^35^, a response which can stimulate N_2_O production^20^. However, a recent meta-analysis suggests that, as far as N_2_O production is concerned, the effect of NT practices on soil moisture is limited to dry ecosystems^36^. Allowing soil moisture content to diverge between treatments as a result of earthworm activity under the conditions of our study might have led to differences that were less representative of field conditions than the current setup.

Finally, our study did not consider treatment effects on CH_4_ emissions. Because the GWP of net CH_4_ fluxes in upland agroecosystems are typically small compared to those of CO_2_ and N_2_O^4^, treatment effects on CH_4_ fluxes play only a minor role in determining the GHG mitigation potential of most cropping systems. Nonetheless, NT practices have been reported to reduce CH_4_ uptake of soil^37^ or have no effect^38^. The presence of earthworms might reduce CH_4_ uptake of a forest soil under field conditions as well^39^, further suggesting that including these fluxes in GWP calculations may slightly reduce the GHG mitigation potential of NT systems.

Our results suggest that the presence of earthworms, typically increased by NT, can increase GHG emissions from NT systems to the same level as in CT systems. Moreover, the effect of earthworm activity on GHG emissions did not diminish over time, suggesting that earthworm activity is an integral and non-transient component of the GHG balance of NT soils. No-tillage management can certainly be beneficial for soils, as it reduces erosion and improves physical properties that can increase the extent to which soil can absorb water^40^. However, our study provides a key to explaining the finding reported by a growing body of literature that NT soils show limited potential for GHG mitigation^12^,^41^,^42^. We present a biological pathway that can (partly) explain this phenomenon. The presence of earthworms, but preferably of all soil biota, should therefore be included in modeling GHG emissions from agricultural soils.

## Methods

### Experimental lay-out

**Figure 1:**
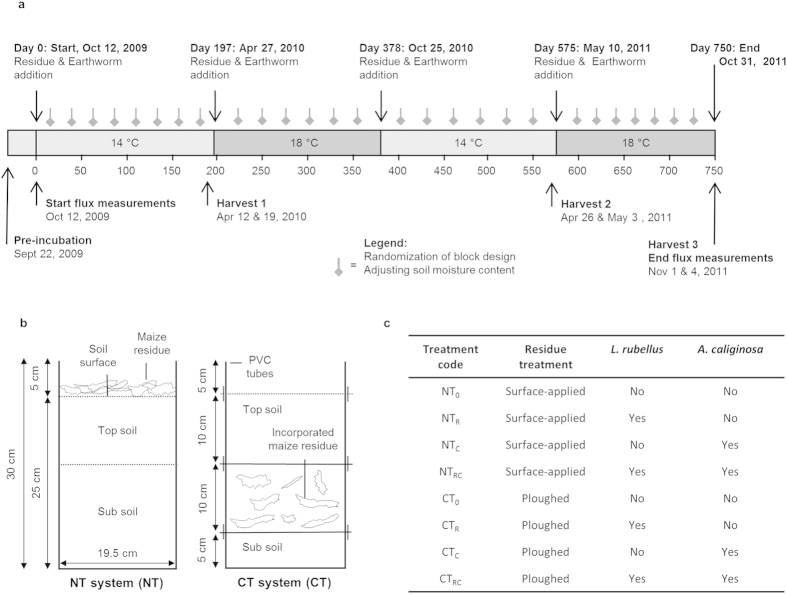
(**a**) Timeline (in days) of the experimental lay-out; (**b**) Experimental mesocosm design; (**c**) Treatment codes.

**Figure 2:**
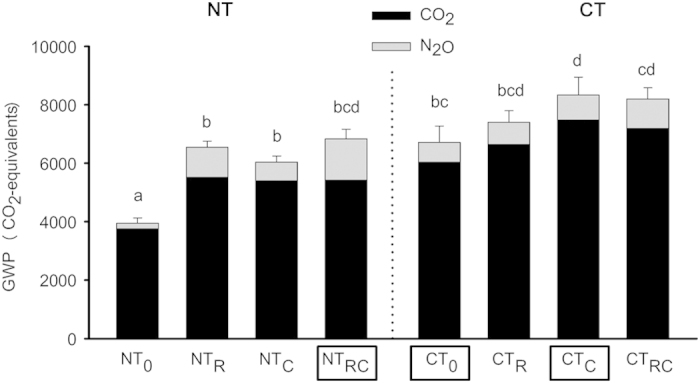
Cumulative (750 days) residue-induced GHG emissions, expressed in terms of GWP, for the simulated NT and CT systems. Error bars denote SEM (*n *= 5). Main effects (ANOVA) for main factors ‘Earthworm presence’ and ‘Tillage treatment’ are *P *< 0.001; their interaction effect is *P *= 0.037. Treatment codes as in Fig. 1c.

**Figure 3:**
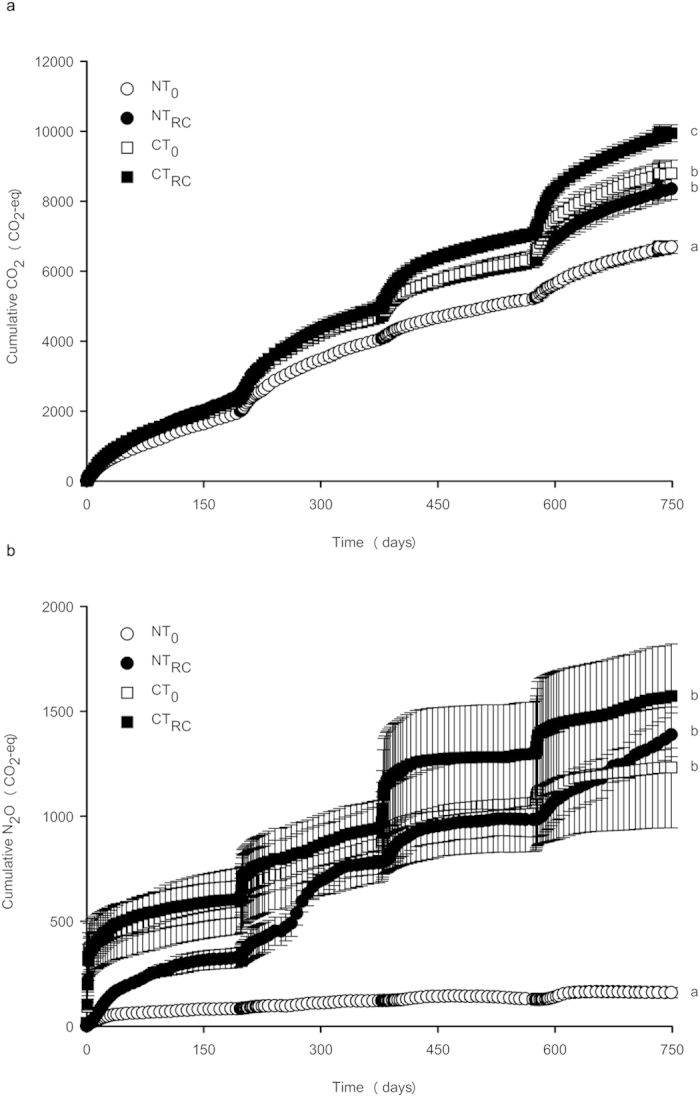
Cumulative CO_2_ (a) and N_2_O emissions (b) during 750 days of incubation. Error bars denote SEM (*n *= 5). Letters indicate significant differences (*P *< 0.05) between treatment means of cumulative N_2_O and CO_2_. Treatment codes as in Fig. 1c.

**Figure 4:**
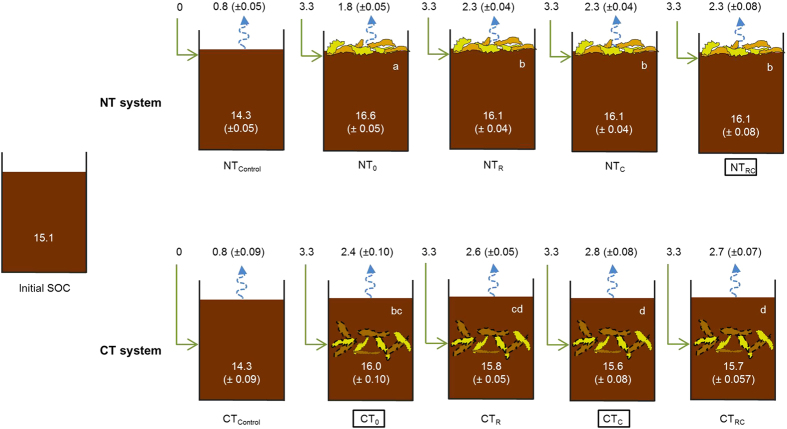
Soil organic carbon (SOC; in g C kg^−1^ soil), cumulative CO_2_ emissions (g C-CO_2_ kg^−1^ soil), and total residue application (g C kg^−1^ soil) after an experimental period of 750 days. SEMs (*n *= 5) are shown in parentheses. Different letters inside the mesocosms indicate differences between treatments, excluding the control. Treatment codes as in Fig. 1c. ANOVA of single-species effects of the earthworms and their interaction on SOC are given in Supplementary Table 5. Total C concentrations have also been measured directly from the top- and subsoil of the mesocosm soil profile (Methods). No changes in C storage through earthworm activity were detected for the topsoil of NT and CT treatments (see also Supplementary Table 5).

In a 750-day mesocosm study, we tested the effects of residue placement (simulating NT and CT), earthworm presence (of the epigeic *Lumbricus rubellus* (Hoffmeister) and the endogeic *Aporrectodea caliginosa (Savigny)*) and their interactions on N_2_O and CO_2_ emissions, as well as on total organic carbon (SOC) content. The study was set up as a full factorial 2 × 2 × 2 design, with tillage treatment (surface-applied residue to simulate an NT system, or residue artificially ploughed into the soil to simulate a CT system), the presence of *L. rubellus* (presence or absence) and the presence of *A. caliginosa* (presence or absence) as independent factors (Fig. 1c). Treatments without residue and earthworms were included as a control (for both the simulated NT and CT system). Treatments were laid out in a randomized block design with five blocks, each containing one replicate of each treatment. Maize (*Zea mays* L.) residues were applied approximately every 190 days (four times in total; see Fig. 1a for a timeline) to mesocosms filled with a loess soil. Applying crop residues to the soil twice a year is common practice in arable farming in the Netherlands; the ploughing-in of crop residues in fall and of cover crops in spring^26^. The study was performed in a climate controlled room at 14 °C after the first and third residue application, and at 18 °C after the second and fourth residue application, to simulate soil temperature variation during the year (Fig. 1a). The relative humidity was 80%. To enable destructive soil analyses and determine earthworm survival during the 750-day span of the experimental period, 10 extra replicates were set-up and distributed over the five blocks; five replicates were harvested after 180 days and the other five after 555 days (Fig. 1a). The study therefore initially consisted of nine treatments with each 15 replicates (135 mesocosms).

### Soil and earthworm collection

The loess soil (*Gleyic Luvisol*, with 20% sand, 61% silt and 19% clay) was collected from the 0–25 cm layer at arable farm ‘Wijnandsrade’ in the South of the Netherlands (50°54′ N, 5°52′ E). The soil contained 15.1 g total C kg^−1^, 1.04 g total N kg^−1^, and had a pH-H_2_O of 6.4. It was sieved through an 8 mm screen, air-dried at 20 °C and repeatedly mixed to ensure homogeneity. To eliminate all earthworm cocoons, the greater part of the soil was treated with γ-irradiation (25 kGy, at Gammaster BV, Ede, the Netherlands). The rest of the soil was sieved through a 2 mm screen to remove earthworm cocoons, but retain propagules of microbes and micro-fauna, and was used as inoculum for the irradiated soil.

Adults and large juveniles of both earthworm species were collected from park areas in Wageningen, the Netherlands, two weeks prior to the start of the experiment or any later earthworm additions. They were stored at 14 °C in plastic containers with loess soil and poplar (*Populus* spp. L.) leaves as feed.

### Set-up of the mesocosms

Every mesocosm had a height of 30 cm and was constructed of one (NT treatments) or four (CT treatments) polyvinyl chloride (PVC) rings (19.5 m inner diameter). This set-up (Fig. 1b) allowed the removal of soil layers for residue incorporation. The four PVC rings were put together with duct tape (poly-ethylene resin and rubber-based adhesive, Wiltec BV, Uden, the Netherlands) to ensure air tightness. The soil profile consisted of a mixture of 7.80 kg of air-dried irradiated soil and 0.40 kg air-dried inoculum (sieved through 2 mm) soil, packed to a bulk density of 1.40 g cm^−3^. The total depth of the soil profile was approximately 25 cm. Gravimetric soil moisture was brought to 275 g water kg^−1^ soil, corresponding to 58% water filled pore space. We checked the average soil moisture content of three to four mesocosms from every block gravimetrically every 2-3 days during the first four weeks of the experimental period, adjusting all mesocosms when necessary. After these four initial weeks we adjusted the average soil water content weekly in a similar manner. We checked each mesocosm gravimetrically when randomizing the block design approximately every four weeks; total soil moisture evaporated from the mesocosms was always less than 5%. After a pre-incubation of 20 days at 14 °C, when N_2_O and CO_2_ emissions had stabilized (see below for gas monitoring procedures), residues and earthworms were added to the mesocosms for the first time. Each mesocosm was covered with a black polyethylene cloth that allowed gaseous exchange with air, decreased water evaporation, and prevented earthworms from escaping.

### Residue and earthworm addition

At every residue application event all treatments received 15 g of maize (*Zea mays* L.) residues, consisting of 13.0 g dry weight of leaves and shoots (6.4 g N kg^−1^, 451.4 g C kg^−1^) and 2.0 g dry weight of roots (4.5 g N kg^−1^, 461.4 g C kg^−1^), chopped in <2 cm pieces. This corresponded to an application rate of approximately 5 Mg dry matter ha^−1^, based on the surface area of the mesocosms (0.030 m^2^). For the NT treatments, we loosened the upper 2 cm of soil surface with a knife before placing the residues on the soil surface to optimize contact between residue and soil. For the CT treatments, we mixed the residues into the soil at 10–20 cm depth by first removing the duct tape that was keeping the four ringed-mesocosms air tight. To realistically simulate the ploughing-in of crop residues, we separated the respective soil layer with a metal sheet and removed the 10 cm ring to incorporate 15 g maize residue by hand. Subsequently we reassembled the rings again with duct tape. When adding maize residue after 197 days, we took the 0–10 cm soil layer, mixed the residues through this layer and placed this layer at 10–20 cm depth. The former 10–20 cm soil layer (with the residues mixed in from the previous residue ploughing event) was placed upside down on top of the new 10–20 cm layer (Fig. 1b). This ‘ploughing-procedure’ was repeated two more times, on day 378 and day 575. The bottom 5 cm of the soil profile (total depth of 25 cm) stayed untouched throughout the experiment.

Along with the residue additions, we also added fresh earthworms to the mesocosms. At the start of the experiment, we added 4 individuals of *L. rubellus* and 7 individuals of *A. caliginosa*, corresponding to 125 and 225 individuals m^−2^, respectively (Supplementary Table 2 lists added earthworm numbers and biomass). These densities are in line with reported values in tillage and pasture systems from various countries and continents^17^,^43^. The number of individuals that were applied in later earthworm additions were based on earthworm survival data retrieved from the first and second harvests, as earthworm mortality increased over the experimental period of 750 days (Supplementary Table 3 for earthworm weight differences after the first and second harvests). Mean percent biomass loss for *L. rubellus* increased from 41% after the first harvest to 99% after the third harvest (*P* < 0.001). For *A. caliginosa* biomass loss increased from 36% to 74% (*P* < 0.001). Before entering the experiment, earthworms were washed and moved to damp filter paper to void gut contents before weighing^44^.

The substantial mortality rate of the earthworms, especially of *L. rubellus*, might have increased the amount of available N in the soil to some extent. However, the amount of earthworm-N that could have become available for denitrification (and thereby N_2_O production) was less than 1% of the total NO_3_^−^ in the mesocosm soil. Shortly, *L. rubellus* has an ash-free dry mass of approx. 6.3% of total weight^45^ and an N content of approx. 8.4% ash-free dry mass^46^. With an average 41% biomass loss of 3.25 g of initially introduced *L. rubellus* after the first harvest (Supplementary Tables 2 and 3), this results in approximately 7 mg earthworm-N per mesocosm. This is less than 1% of the approximately 720 mg N-NO_3_^−^ present in the mesocosm soil (Supplementary Table 5). Hence, it is unlikely that the death of individuals of *L. rubellus* can explain increased fluxes of N_2_O emission.

### N_2_O and CO_2_ flux measurements and calculations

Flux measurements of N_2_O and CO_2_ were taken daily during the first 5 days after every residue application, every second day in week 2 and 3, every third day in week 4–6, and once a week until the next residue application or the end of the experiment (153 flux measurements in 750 days). The flux measurement protocol largely followed that of previous studies^34^,^47^ and was in agreement with good measurement practices as formulated by Rosenstock *et al.*^42^ and Igbal *et al.*^48^,^49^. Polypropylene flux chambers equipped with two rubber septa were placed on the mesocosm for approximately 30 minutes. Gas measurements were taken with the INNOVA 1312 Photo-acoustic Multi Gas Monitor by INNOVA Air Tech Instruments, Ballerup, Denmark, using an external soda-lime filter to minimize interference by CO_2_^50^. The following filters were installed: UA0983 for CO_2_, UA0985 for N_2_O, and SB0527 for water vapor. The CO_2_ and N_2_O filters were in positions A and B of the filter carousel, respectively. Positions D and E were vacant, and the water vapor filter was in position W. The INNOVA 1312 was pre-calibrated by the manufacturer using NIST traceable calibration gases. A full calibration of the optical filters for CO_2_ and N_2_O, involving zero point calibration (using zero gas or pure nitrogen), humidity-interference calibration (using water vapor), span calibration, using a known concentration of CO_2_ [10000 ppm] and N_2_O [1.0 ppm], and cross-interference calibration, was done in 2009 (ENMO-Bruel & Kjaer Sound and Vibration Technology, Turnhout, Belgium). The detection limits for PCO_2_ and PN_2_O estimated as vol. ppm at 2 °C and 1 atm. and SIT = 5, at constant water vapor below 7000 mg m^−3^, were 3.4 and 0.03, respectively. Gas fluxes were calculated by assuming a linear increase of gas concentration over time. Cumulative emissions were calculated by assuming linear changes between subsequent flux measurements^51^.

## Calculations

To calculate the effect of earthworm activity on the net GWP balance, we followed Lubbers *et al.* (2013)^16^. In short, we transformed values for CO_2_ and N_2_O to CO_2_ equivalents (CO_2_-eq)^25^, using a 100-year time horizon as in the Kyoto Protocol, and expressed the contributions of N_2_O-N (CO_2_-eq-N_2_O) and CO_2_-C (CO_2_-eq-CO_2_) as % of the net GWP.

The change in SOC during the experimental period of 750 days was calculated based on the balance between C input (residue) and output (CO_2_ flux). The initial SOC content for all treatment combinations was 15.1 g C kg^−1^ soil. Maize residue applications amounted to 3.3 g C kg^−1^ soil, except for the control treatments. The control treatments did not receive any added C from residues. Since the mesocosm set-up did not allow for leaching SOC or for acquiring C through photosynthesis, changes in SOC after the experimental period of 750 days could be calculated by subtracting the amount of C in the cumulative CO_2_ emissions from the initial SOC content and the C from the added maize residues.

### Soil analysis

Gravimetric soil moisture content and bulk density (BD) were determined at all three harvest dates. Samples for the determination of BD were taken from two sampling depths (intact soil core samples (100 cm^3^) at 5–10 cm from the 0–10 cm ‘topsoil’, and at 15–20 cm from the 10–25 cm ‘subsoil’), because the effects of earthworm functional groups on soil compaction might occur at different profile depths (Supplementary Table 4). Representative subsamples at equal depths were taken for pH and mineral N analysis. Nitrate and nitrite (NO_3_-N + NO_2_-N) and ammonium (NH_4_-N) concentrations, and pH (all in 0.01 M CaCl_2_) were determined only in the mesocosms of the first harvest; further analysis was redundant since nitrate and ammonium concentrations were high (far from limiting microbial N processes like nitrification and denitrification) and there were no differences between treatments (Supplementary Table 5). Total C in the top- and subsoil was determined only in the mesocosms of the final harvest (Supplementary Table 6). Subsamples were ball-milled and oven-dried at 60 °C and approximately 40 mg was weighed out in tin cups, the precise weight was recorded, and the samples were sent to the Stable Isotope Facility of UC Davis for measurement of total C in a PDZ Europa ANCA-GSL elemental analyser (Sercon Ltd, Crewe, Cheshire, UK). The C content was considered to be exclusively organic C, as there were no carbonates present in the loess soil.

Simultaneously with soil sampling, the mesocosms were carefully disassembled and earthworms were collected. The numbers of surviving earthworms were recorded per species, and fresh weights were determined after the gut contents had been voided following the method mentioned above.

### Statistical analysis

Analysis of variance was performed using the general ANOVA module in SPSS (IBM SPSS Statistics 19.0). Gas emission data and soil parameters were analyzed using a two-way ANOVA with blocking, with the three independent factors being tillage treatment (NT or CT), the presence or absence of *L. rubellus* and the presence or absence of *A. caliginosa*. For further analysis of the effects of earthworms, gas emission data and soil parameters were analyzed for each tillage treatment separately (the simulated NT and the CT systems), the two independent factors being the presence or absence of *L. rubellus* and the presence or absence of *A. caliginosa*. We assessed significant differences in treatment means by using ANOVA and post hoc (Tukey) analysis at 95% confidence. Earthworm survival data were analyzed with one-way ANOVAs with blocking and the presence of either *L. rubellus* (in case of *A. caliginosa* survival) or *A. caliginosa* (in case of *L. rubellus* survival) as the independent factor.

## Additional Information

**How to cite this article**: Lubbers, I. M. *et al.* Reduced greenhouse gas mitigation potential of no-tillage soils through earthworm activity. *Sci. Rep.* **5**, 13787; doi: 10.1038/srep13787 (2015).

## Supplementary Material

Supplementary Information

## Figures and Tables

**Table 1:** Source of variation (ANOVA) for two statistical models for the cumulative GWP, CO_2_ and N_2_O emissions.

Source of variation	Day 0–197			Day 0–378			Day 0–575			Day 0–750		
	GWP	CO_2_	N_2_O	GWP	CO_2_	N_2_O	GWP	CO_2_	N_2_O	GWP	CO_2_	N_2_O
**Model I:**												
Tillage treatment	<0.001	<0.001	<0.001	<0.001	<0.001	0.026	<0.001	<0.001	0.039	<0.001	<0.001	0.316
Earthworm presence	0.001	<0.001	0.199	<0.001	<0.001	0.020	<0.001	<0.001	0.013	<0.001	<0.001	0.004
Tillage treatment x Earthworm presence	0.017	0.008	0.300	0.003	0.005	0.028	0.058	0.049	0.169	0.037	0.078	0.063
Block	0.003	<0.001	0.023	0.002	<0.001	0.049	0.014	0.001	0.060	0.020	0.002	0.125
**Model II:**												
Tillage treatment	<0.001	<0.001	<0.001	<0.001	<0.001	0.139	<0.001	<0.001	0.097	<0.001	<0.001	0.946
*L. rubellus*	0.028	0.065	0.119	<0.001	0.001	<0.001	<0.001	<0.001	0.008	<0.001	0.001	0.002
*A. caliginosa*	0.022	0.002	0.583	0.004	<0.001	0.240	0.001	<0.001	0.100	<0.001	<0.001	0.025
*L. rubellus* x *A. caliginosa*	0.121	0.015	0.965	0.055	0.001	0.679	0.024	<0.001	0.906	0.010	<0.001	0.992
Tillage treatment x *L. rubellus*	0.005	0.001	0.297	0.002	0.003	0.015	0.021	0.026	0.065	0.006	0.012	0.015
Tillage treatment x *A. caliginosa*	0.294	0.499	0.371	0.273	0.649	0.160	0.792	0.896	0.625	0.968	0.429	0.465
Tillage treatment x *L. rubellus* x *A. caliginosa*	0.774	0.480	0.845	0.232	0.130	0.657	0.489	0.206	0.934	0.308	0.133	0.778
Block	0.003	<0.001	0.031	0.002	0.001	0.023	0.015	0.002	0.054	0.015	0.001	0.086

After each residue addition the emission data have been cumulatively calculated, resulting into four experimental time spans that last approx. 180-200 days longer each time. Model I includes two main factors, ‘Tillage treatment (NT or CT)’ and ‘Earthworm presence (yes or no)’, and their interaction, as well as the significance of variation assigned to the block effect. Model II includes three main factors, ‘Tillage treatment (NT or CT)’, ‘*L. rubellus* (yes or no)’, and ‘*A. caliginosa* (yes or no)’, and their interactions, as well as the significance of variation assigned to the block effect.

**Table 2:** Cumulative GWP, CO_2_ and N_2_O, expressed in CO_2_-equivalents, from simulated NT (no-tillage) and CT (conventional tillage) systems for the presence of *L. rubellus* and *A. caliginosa*, separately and in combination.

Treatment	GWP	Day 0–197		GWP	Day 0–378		GWP	Day 0–575		GWP	Day 0–750	
		CO_2_	N_2_O		CO_2_	N_2_O		CO_2_	N_2_O		CO_2_	N_2_O
**No-tillage (NT):**												
NT_0_	2016 (±73)	1934 (±72)	82 (±14)	4159 (±124)	4037 (±123)	121 (±14)	5338 (±130)	5210 (±129)	127 (±18)	6848 (±186)	6689 (±184)	160 (±24)
NT_R_	2608 (±82)	2377 (±80)	230 (±20)	5540 (±95)	4948 (±78)	591 (±55)	7098 (±127)	6378 (±102)	720 (±76)	9452 (±165)	8441 (±141)	1010 (±86)
NT_C_	2457 (±43)	2297 (±38)	160 (±19)	5083 (±58)	4771 (±52)	312 (±26)	6757 (±134)	6324 (±127)	433 (±43)	8945 (±179)	8328 (±156)	616 (±89)
NT_RC_	2740 (±105)	2414 (±94)	326 (±48)	5670 (±142)	4892 (±130)	778 (±55)	7294 (±218)	6310 (±202)	984 (±82)	9734 (±319)	8346 (±302)	1388 (±103)
**ANOVA**												
*L. rubellus*	<0.001***	<0.001***	<0.001***	<0.001***	<0.001***	<0.001***	<0.001***	<0.001***	<0.001***	<0.001***	<0.001***	<0.001***
*A. caliginosa*	0.002**	0.003**	0.007**	<0.001***	0.001**	<0.001***	<0.001***	0.001**	<0.001***	<0.001***	<0.001***	<0.001***
*L. rubellus* x	0.051	0.010*	0.740	<0.001***	<0.001***	0.957	<0.001***	0.626	<0.001**	<0.001***	0.599	
*A. caliginosa*												
Block	0.105	0.017*	0.233	0.158	0.036*	0.267	0.288	0.048*	0.058	0.151	0.045*	0.162
**Conventional tillage (CT):**												
CT_0_	2877 (±259)	2283 (±173)	594 (±157)	5540 (±343)	4677 (±176)	863 (±181)	6851 (±368)	5887 (±213)	1064 (±233)	10026 (±559)	8793 (±380)	1233 (±288)
CT_R_	2926 (±125)	2285 (±112)	640 (±116)	5758 (±198)	4857 (±92)	901 (±140)	7502 (±312)	6364 (±104)	1137 (±222)	10715 (±395)	9398 (±195)	1317 (±244)
CT_C_	3095 (±209)	2508 (±95)	587 (±144)	5888 (±347)	5104 (±199)	784 (±170)	7829 (±470)	6648 (±211)	1181 (±298)	11646 (±616)	10231 (±304)	1415 (±318)
CT_RC_	2930 (±213)	2325 (±43)	605 (±88)	5913 (±281)	4968 (±202)	945 (±131)	7836 (±365)	6539 (±193)	1297 (±246)	11513 (±381)	9940 (±245)	1573 (±248)
**ANOVA**												
*L. rubellus*	0.534	0.299	0.626	0.390	0.843	0.194	0.227	0.175	0.401	0.377	0.470	0.381
*A. caliginosa*	0.245	0.139	0.751	0.090	0.031*	0.815	0.021*	0.003**	0.274	0.002**	0.001**	0.127
*L. rubellus* x												
*A. caliginosa*	0.264	0.289	0.829	0.493	0.175	0.409	0.219	0.040*	0.974	0.200	0.055	0.788
Block	<0.001***	0.017*	<0.001***	< 0.001***	0.004**	<0.001***	0.002**	0.009**	<0.001***	0.002**	0.018*	<0.001***

SEMs are shown in parentheses (*n *= 5). Levels of significance: *<0.05; **<0.01; ***<0.001. After each residue addition the emission data have been cumulatively calculated, resulting into four experimental time spans that last approx. 180–200 days longer each time. Treatment codes as in Fig. 1c.
