# Supplementary material for: Reduced greenhouse gas mitigation potential of no-tillage soils through earthworm activity
Source: Sci Rep. 2015 Sep 4;5:13787. doi: 10.1038/srep13787 (PMC4642549; doi:10.1038/srep13787)
Supplement: Supplementary Information [file srep13787-s1.doc]

**Reduced greenhouse gas mitigation potential of no-tillage soils through earthworm activity**

**Supplementary Information**

Ingrid M. Lubbers^1*^, Kees Jan van Groenigen^2^, Lijbert Brussaard^1^ and Jan Willem van Groenigen^1^

*^1^Department of Soil Quality, Wageningen University, PO BOX 47, 6700AA Wageningen, the Netherlands.* ^2^*Center for Ecosystem Science and Society, Department of Biological Sciences, Northern Arizona University, Flagstaff, Arizona 86011, USA.*

*e-mail: [ingrid.lubbers@wur.nl](mailto:ingrid.lubbers@wur.nl)

**Supplementary Tables**

**Supplementary Table 1** │ **Earthworm fresh weight introduced in four earthworm additions on day 1, 197, 378 and 575 of the experimental period.**

| **Treatment** | **Earthworm weight introduced per mesocosm (g)** | | | | | | | |
| --- | --- | --- | --- | --- | --- | --- | --- | --- |
|  | Day 0 | | Day 197 | | Day 378 | | Day 575 | |
|  | *L. rubellus*  (4 individuals)  *n* = 15 | *A. caliginosa*  (7 individuals)  *n* = 15 | *L. rubellus*  (2 individuals)  *n* = 10 | *A. caliginosa*  (2 individuals)  *n* = 10 | *L. rubellus*  (2 individuals)  *n* = 10 | *A. caliginosa*  (2 individuals)  *n* = 10 | *L. rubellus*  (4 individuals)  *n* = 5 | *A. caliginosa*  (5 individuals)  *n* = 5 |
| NT_0_ |  |  |  |  |  |  |  |  |
| NT _R_ | 3.08 (±0.10) |  | 2.04 (±0.09) |  | 2.52 (±0.17) |  | 3.25 (±0.19) |  |
| NT _C_ |  | 5.21 (±0.15) |  | 1.29 (±0.04) |  | 1.46 (±0.06) |  | 2.55 (±0.17) |
| NT _RC_ | 3.42 (±0.20) | 5.11 (±0.16) | 2.17 (±0.07) | 1.16 (±0.04) | 2.40 (±0.09) | 1.45 (±0.07) | 3.05 (±0.14) | 2.52 (±0.17) |
| CT_0_ |  |  |  |  |  |  |  |  |
| CT _R_ | 3.24 (±0.13) |  | 2.12 (±0.07) |  | 2.47 (±0.12) |  | 3.04 (±0.07) |  |
| CT _C_ |  | 5.34 (±0.13) |  | 1.31 (±0.04) |  | 1.55 (±0.07) |  | 2.66 (±0.11) |
| CT _RC_ | 3.32 (±0.21) | 5.18 (±0.15) | 2.03 (±0.06) | 1.12 (±0.02) | 2.53 (±0.21) | 1.49 (±0.06) | 2.97 (±0.07) | 2.50 (±0.08) |

SEMs are shown in parentheses. Treatment codes as in Fig. 1c.

**Supplementary Table 2 │** Earthworm fresh weight differences during the course of the experiment after 180, 555 and 750 days.

| **Treatment** | **Biomass loss, %** | | | | | |
| --- | --- | --- | --- | --- | --- | --- |
|  | Day 0-180 | | Day 180 - 555 | | Day 555 - 750 | |
|  | *L. rubellus* | *A. caliginosa* | *L. rubellus* | *A. caliginosa* | *L. rubellus* | *A. caliginosa* |
| **No-tillage (NT)** |  |  |  |  |  |  |
| NT _R_ | 47.9 (±13.7) |  | 93.8 (± 6.2) |  | 100.0 (±0.0) |  |
| NT _C_ |  | 40.9 (±3.0) |  | 57.3 (±7.8) |  | 68.7 (±7.0) |
| NT _RC_ | 33.5 (±13.5) | 42.5 (±1.9) | 94.4 (± 3.9) | 85.9 (±4.7) | 100.0 (±0.0) | 91.2 (±3.9) |
|  |  |  |  |  |  |  |
| ANOVA |  |  |  |  |  |  |
| *L. rubellus* |  | 0.777 |  | 0.010* |  | 0.042* |
| *A. caliginosa* | 0.461 |  | 0.919 |  | No value |  |
| *Block* | 0.583 | 0.977 | 0.221 | 0.144 | No value | 0.452 |
|  |  |  |  |  |  |  |
| **Conventional tillage (CT)** |  |  |  |  |  |  |
| CT _R_ | 27.9 (±2.4) |  | 93.5 (±2.7) |  | 98.8 (±1.2) |  |
| CT _C_ |  | 33.4 (±4.8) |  | 64.1 (±7.1) |  | 66.7 (±0.9) |
| CT _RC_ | 51.5 (±8.4) | 29.0 (±6.9) | 96.9 (±3.1) | 68.3 (±2.9) | 98.1 (±1.5) | 71.6 (±2.7) |
|  |  |  |  |  |  |  |
| ANOVA |  |  |  |  |  |  |
| *L. rubellus* |  | 0.806 |  | 0.668 |  | 0.220 |
| *A. caliginosa* | 0.049* |  | 0.523 |  | 0.740 |  |
| *Block* | 0.475 | 0.265 | 0.895 | 0.866 | 0.727 | 0.669 |

SEMs are shown in parentheses (*n* = 5). Levels of significance: * < 0.05; ** < 0.01; *** < 0.001. Treatment codes as in Fig. 1c.

**Supplementary Table 3 │ Bulk density for top- and subsoil at harvests 1, 2 and 3.**

| **Treatment** | **† Bulk density (g cm^-3^) at harvest 1, 2 and 3** | | | | | |
| --- | --- | --- | --- | --- | --- | --- |
|  | Harvest 1, April 12 & 19, 2010 | | Harvest 2, April 26 & May 3, 2011 | | Harvest 3, November 1 & 4, 1011 | |
|  | Topsoil  (0 – 10 cm) | Subsoil  (10 – 25 cm) | Topsoil  (0 – 10 cm) | Subsoil  (10 – 25 cm) | Topsoil  (0 – 10 cm) | Subsoil  (10 – 25 cm) |
| **No-tillage (NT):** |  |  |  |  |  |  |
| NT_0_ | 1.33 (±0.01) | 1.39 (±0.01) | 1.25 (±0.02) | 1.26 (±0.01) | 1.23 (±0.02) | 1.28 (±0.01) |
| NT _R_ | 1.35 (±0.02) | 1.37 (±0.01) | 1.24 (±0.01) | 1.28 (±0.01) | 1.22 (±0.02) | 1.30 (±0.02) |
| NT _C_ | 1.39 (±0.02) | 1.39 (±0.01) | 1.28 (±0.01) | 1.26 (±0.02) | 1.28 (±0.02) | 1.29 (±0.02) |
| NT _RC_ | 1.33 (±0.03) | 1.38 (±0.01) | 1.26 (±0.01) | 1.27 (±0.02) | 1.28 (±0.02) | 1.28 (±0.02) |
|  |  |  |  |  |  |  |
| **ANOVA** |  |  |  |  |  |  |
| *L. rubellus* | 0.210 | 0.180 | 0.203 | 0.438 | 0.914 | 0.926 |
| *A. caliginosa* | 0.210 | 0.180 | 0.089 | 0.517 | 0.018* | 0.853 |
| *L. rubellus x A. caliginosa* | 0.024* | 0.594 | 0.742 | 0.794 | 0.829 | 0.410 |
| *Block* | 0.052 | 0.150 | 0.428 | 0.528 | 0.437 | 0.760 |
|  |  |  |  |  |  |  |
| **Conventional tillage (CT):** |  |  |  |  |  |  |
| CT_0_ | 1.35 (±0.02) | 1.44 (±0.01) | 1.28 (±0.02) | 1.27 (±0.03) | 1.16 (±0.02) | 1.26 (±0.02) |
| CT _R_ | 1.37 (±0.01) | 1.41 (±0.01) | 1.26 (±0.00) | 1.28 (±0.01) | 1.18 (±0.01) | 1.27 (±0.02) |
| CT _C_ | 1.35 (±0.01) | 1.31 (±0.02) | 1.26 (±0.01) | 1.34 (±0.02) | 1.22 (±0.02) | 1.35 (±0.01) |
| CT _RC_ | 1.39 (±0.03) | 1.41 (±0.02) | 1.31 (±0.02) | 1.35 (±0.02) | 1.22 (±0.02) | 1.35 (±0.01) |
|  |  |  |  |  |  |  |
| **ANOVA** |  |  |  |  |  |  |
| *L. rubellus* | 0.130 | 0.035* | 0.416 | 0.559 | 0.641 | 0.868 |
| *A. caliginosa* | 0.779 | 0.001** | 0.416 | 0.003** | 0.022* | 0.000*** |
| *L. rubellus x A. caliginosa* | 0.641 | 0.002** | 0.073 | 0.873 | 0.561 | 0.781 |
| *Block* | 0.323 | 0.480 | 0.828 | 0.380 | 0.295 | 0.480 |

SEMs are shown in parentheses (*n* = 5). Levels of significance: * < 0.05; ** < 0.01; *** < 0.001. Treatment codes as in Fig. 1c.

† The initial bulk density at the start of the experiment was set at 1.40 g cm^-3^.

**Supplementary Table 4 │ Nitrate and ammonium concentrations and pH for top- and subsoil at harvest 1, on April 12 and 19.**

| **Treatment** | **Mineral N and pH from 0.01 m CaCl_2_ at harvest 1** | | | | | |
| --- | --- | --- | --- | --- | --- | --- |
|  | NO_3_^-^ (mg N kg^-1^) | | NH_4_^+^ (mg N kg^-1^) | | pH (CaCl_2_) | |
|  | Topsoil  (0 – 10 cm) | Subsoil  (10 – 25 cm) | Topsoil  (0 – 10 cm) | Subsoil  (10 – 25 cm) | Topsoil  (0 – 10 cm) | Subsoil  (10 – 25 cm) |
| **No-tillage (NT):** |  |  |  |  |  |  |
| NT_0_ | 89.0 (±12.6) | 92.9 (±12.0) | 6.4 (±1.0) | 6.1 (±0.7) | 6.4 (±0.1) | 6.3 (±0.1) |
| NT _R_ | 108.8 (±9.6) | 71.1 (±13.8) | 4.3 (±0.6) | 4.1 (±0.6) | 6.5 (±0.1) | 6.5 (±0.1) |
| NT _C_ | 100.4 (±4.2) | 90.8 (±8.9) | 4.5 (±0.9) | 4.3 (±0.9) | 6.4 (±0.1) | 6.4 (±0.1) |
| NT _RC_ | 93.0 (±16.0) | 87.0 (±6.7) | 5.6 (±1.0) | 5.0 (±0.8) | 6.4 (±0.1 ) | 6.4 (±0.1) |
|  |  |  |  |  |  |  |
| **ANOVA** |  |  |  |  |  |  |
| *L. rubellus* | 0.866 | 0.645 | 0.195 | 0.118 | 0.732 | 0.309 |
| *A. caliginosa* | 0.747 | 0.875 | 0.419 | 0.319 | 0.089 | 0.385 |
| *L. rubellus x A. caliginosa* | 0.751 | 0.293 | 0.027* | 0.386 | 0.613 | 0.584 |
| *Block* | 0.784 | 0.090 | 0.015* | 0.695 | 0.253 | 0.378 |
|  |  |  |  |  |  |  |
| **Conventional tillage (CT):** |  |  |  |  |  |  |
| CT_0_ | 110.6 (±12.3) | 93.1 (±17.0) | 4.1 (±1.1) | 4.2 (±0.7) | 6.4 (±0.1) | 6.4 (±0.0) |
| CT _R_ | 82.1 (±7.7) | 84.4 (±13.4) | 5.7 (±1.0) | 5.8 (±1.2) | 6.3 (±0.1) | 6.5 (±0.1) |
| CT _C_ | 99.7 (±12.6) | 49.5 (±7.9) | 5.7 (±1.0) | 6.2 (±1.2) | 6.4 (±0.1) | 6.7 (±0.1) |
| CT _RC_ | 89.6 (±9.0) | 84.7 (±14.9) | 5.2 (±0.8) | 5.6 (±1.1) | 6.3 (±0.1) | 6.5 (±0.1) |
|  |  |  |  |  |  |  |
| **ANOVA** |  |  |  |  |  |  |
| *L. rubellus* | 0.221 | 0.174 | 0.255 | 0.919 | 0.152 | 0.004** |
| *A. caliginosa* | 0.649 | 0.168 | 0.422 | 0.448 | 0.614 | 0.007** |
| *L. rubellus x A. caliginosa* | 0.533 | 0.108 | 0.288 | 0.185 | 0.282 | 0.504 |
| *Block* | 0.258 | 0.169 | 0.573 | 0.693 | 0.484 | 0.121 |

SEMs are shown in parentheses (*n* = 5). Levels of significance: * < 0.05; ** < 0.01; *** < 0.001. Treatment codes as in Fig. 1c.

**Supplementary Table 5 │ Total C concentrations for top- and subsoil after 750 days.**

| **Treatment** | **Total C after 750 days – direct measurements** | | **Total C after 750 days – calculated values from Fig. 4** |
| --- | --- | --- | --- |
|  | C concentration (g C kg^-1^) | | C concentration (g C kg^-1^) |
|  | Topsoil  (0 – 10 cm) | Subsoil  (10 – 25 cm) | Mesocosm soil  (0 – 25 cm) |
| **No-tillage (NT):** |  |  |  |
| NT_0_ | 13.96 (±0.26) | 14.16 (±0.65) | 16.58 (±0.05) |
| NT _R_ | †15.03 (±0.62) | 13.58 (±0.95 ) | 16.10 (±0.04) |
| NT _C_ | 16.80 (±1.59) | 12.56 (±0.62) | 16.13 (±0.04) |
| NT _RC_ | 14.62 (±0.68) | 14.78 (±1.18) | 16.12 (±0.08) |
|  |  |  |  |
| **ANOVA** |  |  |  |
| *L. rubellus* | 0.724 | 0.331 | < 0.001*** |
| *A. caliginosa* | 0.348 | 0.735 | < 0.001*** |
| *L. rubellus x A. caliginosa* | 0.120 | 0.196 | < 0.001*** |
| *Block* | 0.823 | 0.380 | 0.045 |
|  |  |  |  |
| **Conventional tillage (CT):** |  |  |  |
| CT_0_ | 14.56 (±0.61) | 14.12 (±0.77) | 16.00 (±0.10) |
| CT _R_ | 15.04 (±0.97) | 14.32 (±0.61) | 15.84 (±0.05) |
| CT _C_ | 14.36 (±0.89 | † 12.13 (±0.34) | 15.61 (±0.08) |
| CT _RC_ | 13.44 (±0.22) | † 14.00 (±0.95) | 15.69 (±0.06) |
|  |  |  |  |
| **ANOVA** |  |  |  |
| *L. rubellus* | 0.565 | 0.039* | 0.482 |
| *A. caliginosa* | 0.173 | 0.123 | 0.001*** |
| *L. rubellus x A. caliginosa* | 0.198 | 0.075 | 0.060 |
| *Block* | 0.040* | 0.020* | 0.018* |

SEMs are shown in parentheses (*n* = 5). Levels of significance: * < 0.05; ** < 0.01; *** < 0.001. Treatment codes as in Fig. 1c.

† An outlier was detected, *P* < 0.05 using the Z-score from Grubbs[^1^](#_ENREF_1). SEMs are shown in parentheses (*n* = 4).
